# Discontinuation of intravenous thrombolysis in acute ischemic stroke

**DOI:** 10.1186/s12883-026-05465-5

**Published:** 2026-10-09

**Authors:** Matthias Krämer, Alexander Sekita, Matthias C. Borutta, Ludwig Singer, Tobias Engelhorn, Bernd Kallmünzer, Stefan Schwab, Stefan T. Gerner, Kosmas Macha

**Affiliations:** 1 Department of Neurology, Universitätsklinikum Erlangen, Friedrich- Alexander-Universität Erlangen-Nürnberg (FAU), Schwabachanlage 6, 91054 Erlangen, Germany; 2 Department of Neuroradiology, Universitätsklinikum Erlangen, Friedrich- Alexander-Universität Erlangen-Nürnberg (FAU), Erlangen, Germany; 3 Department of Neurology, m&i Fachklinik Herzogenaurach, Herzogenaurach, Germany

**Keywords:** Ischemic stroke, Intravenous thrombolysis, IVT, Alteplase, Rt-PA, Discontinuation, Termination, Interruption, Safety, Intracranial hemorrhage

## Abstract

**Background:**

Alteplase (rt-PA) was the standard thrombolytic in acute ischemic stroke (AIS) for decades. Due to its short half-life it must be administered over 1 h after an initial bolus, but the administration can be stopped when serious side effects occur. However, there are limited data about the frequency and reasons for interrupted or stopped intravenous thrombolysis (IVT) in AIS.

**Methods:**

We retrospectively analyzed our stroke registry over a time of more than six years (January 1, 2018 – March 31, 2024) to determine the proportion of discontinued IVT. We compared the baseline, stroke and treatment characteristics between discontinued and non-discontinued patients. We investigated the reasons for discontinuation and followed up patients who had discontinued treatment to detect any treatment-related complications.

**Results:**

Total 1,504 patients underwent IVT with rt-PA and the treatment was discontinued in 23 patients (1.5%). Of these, 5 patients (0.3%) had their treatment interrupted and 18 patients (1.2%) had their treatment stopped early. On average 61% of the planned dosage was administered in the discontinued patients. The most common reasons for discontinuation were extracranial bleedings, reevaluation of the acute cerebral imaging and deterioration of the neurological symptoms incl. patient agitation. In 7 patients the discontinuation was due to adverse effects of rt-PA, but no patient suffered a symptomatic intracranial hemorrhage. The proportion of additional endovascular thrombectomy was higher in patients with discontinuation than in patients without (47.8% vs. 27.9%; *p* = 0.035).

**Conclusions:**

In our cohort, discontinuation of IVT in the treatment of acute ischemic stroke was rare and the quantity of not administered rt-PA was low.

**Trial registration:**

N.a.

**Supplementary Information:**

The online version contains supplementary material available at https://doi.org/10.1186/s12883-026-05465-5.

## Background

Intravenous thrombolysis (IVT) is the standard therapy for acute ischemic stroke (AIS) [1]. Up to the approval of tenecteplase (TNK) by the European Medicines Agency (EMA) in 2024, alteplase (rt-PA) was the only approved thrombolytic for AIS for decades. Meta-analysis showed a superiority of TNK for excellent functional outcome and recanalization rates of large vessel occlusions (LVO), while confirming a similar safety profile [2–5]. Due to a low half-life of 3.5 min, alteplase must be administered as a bolus followed by a one-hour infusion [6]. In contrast TNK is administered as a bolus over 5–10 s, which is simplifying and speeding up the processes in the treatment of AIS [7, 8]. On the other hand, it is possible to terminate the infusion of rt-PA at any time if serious side effects occur. The frequency of interrupted or stopped rt-PA infusions is not well investigated.

In this study we investigate the proportion and reasons of discontinuation of IVT with rt-PA in our high-volume stroke center in Germany over a period of more than 6 years (01/2018-03/2024) and follow the clinical course of the patients with IVT discontinuation focused on IVT associated complications.

## Methods

### Study design, data acquisition and participants

This retrospective cohort study included AIS patients undergoing IVT with rt-PA at the Department of Neurology, Universitätsklinikum Erlangen, from January 2018 to March 2024. Data were obtained from our local prospective IVT registry including demographic data and pre-stroke condition, antithrombotic pre-medication, clinical and neuroimaging-based stroke characteristics, treatment times for IVT and endovascular thrombectomy (EVT) status. For all patients with interrupted or terminated IVT after the bolus administration, reasons for the discontinuation were obtained from the documentation of the emergency department and occurrence and clinical course of complications were recorded. Patients were generally treated with IVT in accordance with international guidelines [9]. According to our institutional treatment algorithm, in patients within 4.5 h from symptom onset decision for IVT based on non-contrast CT with immediate start of IVT before supplementary CT-Angiography and -Perfusion. Patients in the unknown or extended time window > 4.5 h were treated with IVT based on multimodal CT- or MR-Imaging as described earlier [10]. Supplementary EVT was performed in patients with LVO immediately following an interdisciplinary decision by the treating neurologist and neuroradiologist. Where possible, combination therapy of IVT and EVT was sought. Patients on oral anticoagulation were eligible for IVT dependent on DOAC plasma levels in patients on DOAC therapy or INR measurement in patients on vitamin-k antagonist therapy according to our institutional treatment algorithm [11, 12]. 

### Investigated parameters

We investigated patient baseline characteristics (age, sex, pre-modified Rankin Scale (mRS) score [13], antithrombotic pre-medication), baseline stroke and treatment characteristics (National Institutes of Health Stroke Scale (NIHSS) score [14] at admission, incidence of large vessel occlusion (LVO, defined as occlusion of the internal carotid artery (ICA), middle cerebral artery (MCA), anterior cerebral artery (ACA), posterior cerebral artery (PCA), vertebral artery (VA) or basilar artery (BA)), door-to needle time (DTNt), symptoms-to-needle time (STNt), treatment time window (≤ 4.5 h vs. >4.5 h or unknown time window), ratio of additional EVT). Main objectives were proportion and reasons for the discontinuation of IVT and administered dose of rt-PA in patients with IVT discontinuation. Further IVT associated complications including extra- and intracranial hemorrhages were investigated in patients with IVT discontinuation. Extracranial hemorrhages were classified according to Bleeding Acedamic Research Consortium (BARC) definitions [15]. Symptomatic intracranial hemorrhage (sICH) was defined as any ICH with neurological deterioration, as indicated by a NIHSS score increase of 4 or more points from baseline or the lowest score, or any hemorrhage leading to death according to European Cooperative Acute Stroke Study II (ECASS II) -criteria [16]. In patients with IVT discontinuation we further investigated NIHSS scores at discharge, mortality during the inpatient stay and EVT results according to the modified Treatment in Cerebral Ischemia Scale (mTICI) [17, 18]. 

### Statistical analysis

Data were presented as absolute (relative) numbers for categorial variables or median (interquartile range) for continuous variables, as appropriate. Statistical comparison between patient groups was performed using the Mann-Whitney *U*, the χ^2^ test, and the Fisher exact test, as appropriate. Statistical significance was set a priori at *P* values < 0.05. Statistical analyses were performed using IBM SPSS Statistics 21 software package (www.spss.com).

## Results

**Fig. 1:**
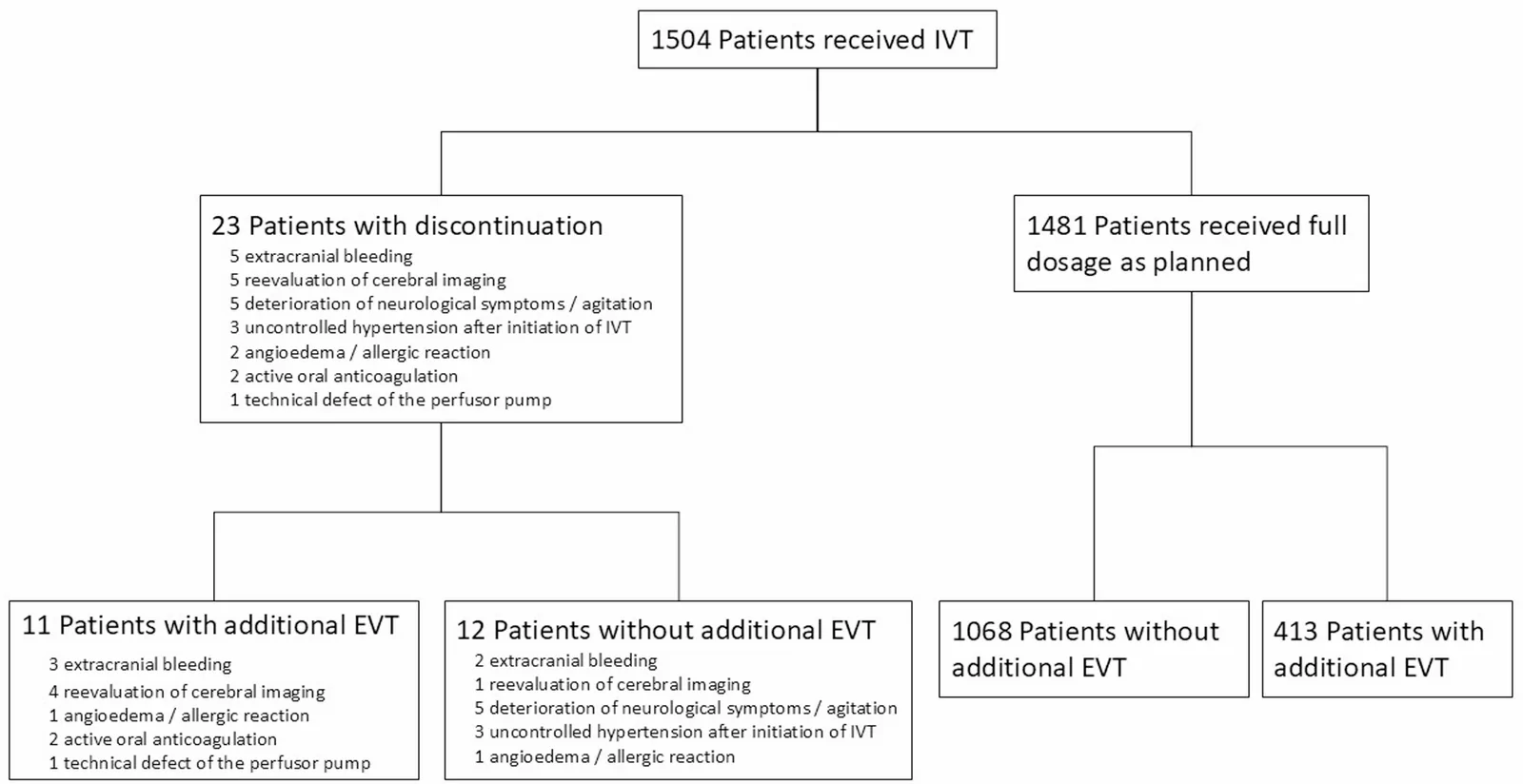
Flow chart of study cohort with indication of reasons for IVT discontinuation

Between January 1, 2018, and March 31, 2024, 1,504 patients presenting with suspected AIS were treated with IVT in the emergency department. 1,481 patients received the full dosage rt-PA without discontinuation (non-DISC) and in 23 patients (1.5%) the treatment was discontinued after the initial bolus or within the 1-hour infusion (DISC). Of these, 5 patients (0.3%) had their treatment interrupted and received the full dose after the interruption, and 18 patients (1.2%) had their treatment stopped early and received a reduced dose. Of the 23 patients with discontinuation of IVT, 11 (47.8%) patients were treated with additional EVT and 12 (52.2%) patients without. (see Fig. 1)

### Comparison of patient baseline characteristics

Patient baseline demographics, stroke characteristics and treatment times are summarized in Table 1. The discontinued patients were younger, more often female and had a lower pre-mRS score but none of these differences were statistically significant. Antithrombotic pre-medication was similar in patient groups DISC and non-DISC.

**Table 1:** Patient baseline and stroke characteristics

	IVT with discontinuation *n* = 23	IVT without discontination *n* = 1481	*p*-value
Age, years median (IQR)	64 (58–82)	77 (65–84)	0.118
**Females**, **n (%)**	13 (56.5)	661 (44.6)	0.255
**pre-mRS score**, **median (IQR)**	0 (0–1)	0 (0–2)	0.067
**Antiplatelet at admission**, **n (%)**	10 (43.5)	548 (37.0)	0.523
**Oral anticoagulation at admission**,** n (%)**	3 (13.0)	148 (10.0)	0.629
**NIHSS score at admission**,** median (IQR)**	9 (4–12)	7 (4–12)	0.532
**Door-to-needle time**,** min median (IQR)**	40 (29–51)	36 (26–53)	0.455
**Symptoms-to-needle time**, **min median (IQR)**	135 (98–265)	155 (105–270)	0.618
**Time window > 4**.**5 h or unknown**,** n (%)**	5 (21.7)	367 (24.8)	0.737
**Large Vessel Occlusion**,** n (%)**	15 (65.2)	679 (45.8)	0.064
**Endovascular Thrombectomy**,** n (%)**	11 (47.8)	413 (27.9)	0.035

### Treatment and stroke characteristics

The median NIHHS score at admission was similar (DISC 9 [IQR 4–12] vs. non-DISC 7 [4–12]; *p* = 0.532). Treatment times represented by the DTNt (DISC 40 min [IQR 29–51] vs. non-DISC 36 min [IQR 26–53]; *p* = 0.455), the STNt (DISC 135 min [IQR 98–265] vs. non-DISC 155 min [IQR 105–270]; *p* = 0.618) and the portion of patients treated in the unknown or extended time window (DISC 21.7% vs. non-DISC 24.8%; *p* = 0.737) were comparable between both groups. The proportion of LVO was higher in the discontinued patients (DISC 65.2% vs. non-DISC 45.8%; *p* = 0.064) but without significance. Discontinued patients received EVT significantly more often (DISC 47.8% vs. non-DISC 27.9%; *p* = 0.035).

### Reasons for discontinued intravenous thrombolysis

Table 2 contains the reasons for discontinuation of IVT. The most common reasons were extracranial bleedings, reevaluation of the acute cerebral imaging and deterioration of the neurological symptoms (increase of NIHSS score ≥ 4 points) incl. agitation leading to cerebral control imaging (each 5 patients; 21.7%). In all five patients with reevaluation of the initial acute cerebral imaging detection of early signs of larger infarcted areas led to the decision to terminate the initiated IVT directly or early after bolus. Other reasons were uncontrolled hypertension (persistently increased systolic blood pressure > 185 mmHg) after initiation of IVT (3 patients; 13.0%; for details see supplementary Table 1), orolingual angioedema / allergic reaction (2 patients; 8.7%), detection of active oral anticoagulation (2 patients; 8.7%) and technical defect of the perfusor pump (1 patient; 4.3%). Therefore in 7 patients (30.4% of discontinued patients; 0.5% of all IVT) the IVT was discontinued due to suspected side effects of rt-PA.

**Table 2:** Reason for discontinuation of IVT

Reason for discontinuation of IVT	*n* (%)
Extracranial bleeding	5 (21.7)
Reevaluation of cerebral imaging	5 (21.7)
Deterioration of neurological symptoms / agitation	5 (21.7)
Uncontrolled hypertension after initiation of IVT	3 (13.0)
Angioedema / allergic reaction	2 (8.7)
Active oral anticoagulation	2 (8.7)
Technical defect of the perfusor pump	1 (4.3)

### Administered dose of rt-PA before discontinuation

**Fig. 2:**
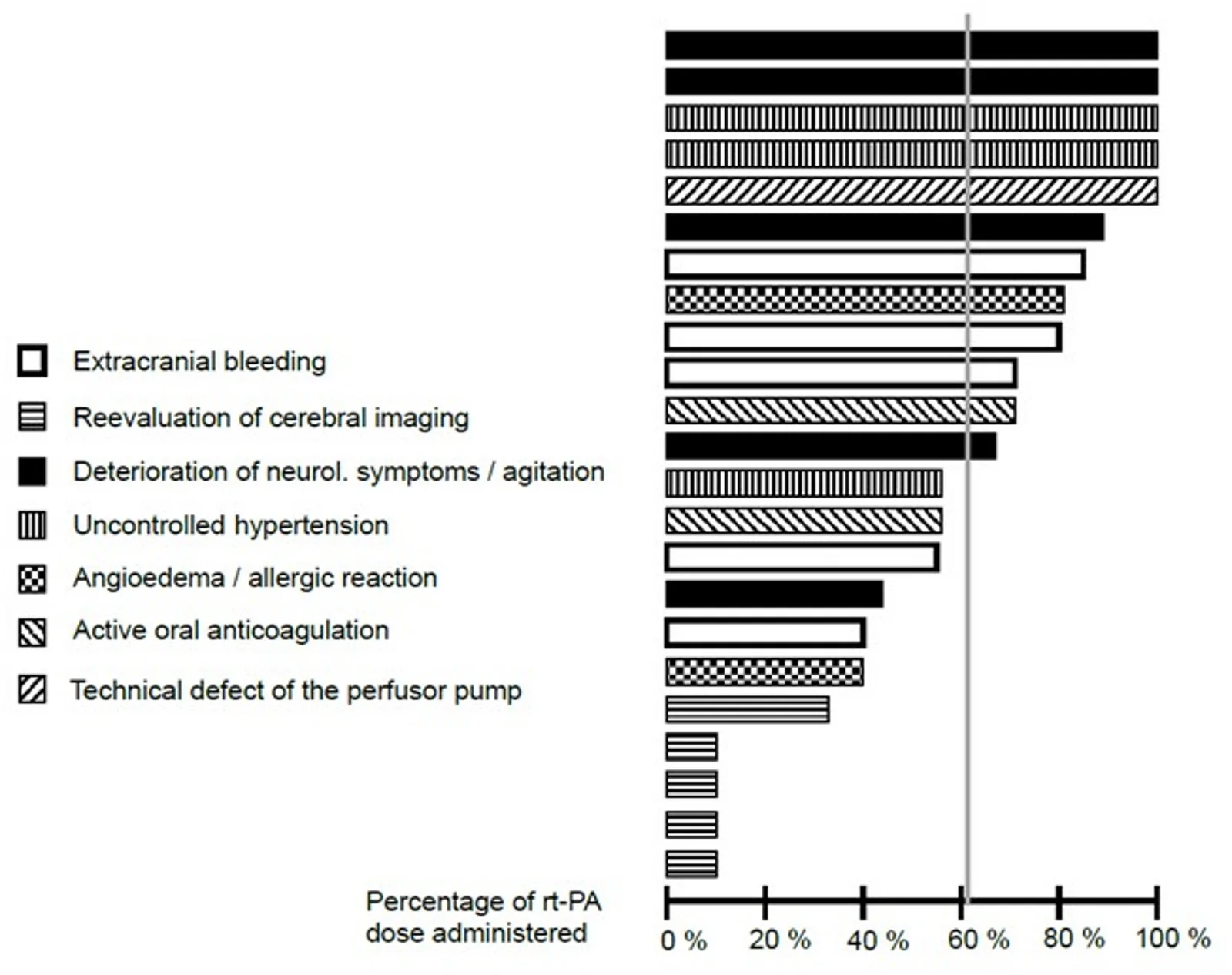
Amount of rt-PA administered by reason for discontinuation

The detailed information of the administered dose for each patient and the complications are listed in Table 3. On average, the discontinued patients received 61% of the planned dosage. Four patients received only the bolus, all of whom underwent reevaluation of the cerebral imaging. The 5 patients with interrupted IVT received the full dose. However, the duration of the interruption remains unclear. Amounts of administered rt-PA by reason for discontinuation is given with Fig. 2.

**Table 3:** Reason and type of discontinuation, rt-PA dose administered and complications in patients with discontinuation of IVT

Reason for discontinuation	Type of discontinuation	Planned full dose of rt-PA	Administered dose of rt-PA	Percentage administered	Any complications (non-ICH)	ICH-complications*
Deterioration of symptoms / agitation	Interruption	90 mg	90 mg	100%	None	None
Deterioration of symptoms / agitation	Interruption	55 mg	55 mg	100%	None	None
Uncontrolled hypertension	Interruption	49 mg	49 mg	100%	None	None
Uncontrolled hypertension	Interruption	45 mg	45 mg	100%	None	Asymptomatic ICH (remote PH)
Technical defect of perfusor pump	Interruption	69 mg	69 mg	100%	None	Asymptomatic ICH (HI1)
Deterioration of symptoms / agitation	Termination	63 mg	56 mg	89%	None	Asymptomatic ICH (HI1)
Extracranial bleeding	Termination	68 mg	58 mg	85%	Post traumatic arm and hip hematoma (BARC type 3a)	None
Angioedema / allergic reaction	Termination	90 mg	73 mg	81%	Angioedema	None
Extracranial bleeding	Termination	50 mg	40 mg	80%	Oral bleeding (BARC type 2)	None
Extracranial bleeding	Termination	70 mg	50 mg	71%	Epistaxis (BARC type 2)	Asymptomatic ICH (SAH)
Active oral anticoagulation	Termination	42 mg	30 mg	71%	None	None
Deterioration of symptoms / agitation	Termination	90 mg	60 mg	67%	None	None
Uncontrolled hypertension	Termination	72 mg	40 mg	56%	None	None
Active oral anticoagulation	Termination	90 mg	50 mg	56%	None	None
Extracranial bleeding	Termination	72 mg	40 mg	56%	Post traumatic periorbital hematoma (BARC type 2)	None
Deterioration of symptoms / agitation	Termination	69 mg	30 mg	44%	None	None
Extracranial bleeding	Termination	Unknown	Unknown	40%**	Epistaxis (BARC type 2)	None
Angioedema / allergic reaction	Termination	63 mg	25 mg	40%	Allergic reaction	None
Reevaluation of cerebral imaging	Termination	70 mg	23 mg	33%	None	None
Reevaluation of cerebral imaging	Termination	90 mg	9 mg	10%	None	Asymptomatic ICH (PH1)
Reevaluation of cerebral imaging	Termination	78 mg	7.8 mg	10%	None	Asymptomatic ICH (SAH)
Reevaluation of cerebral imaging	Termination	72 mg	7.2 mg	10%	None	None
Reevaluation of cerebral imaging	Termination	40 mg	4 mg	10%	None	None

* According to ECASS-II-criteria; ** Calculated based on infusion time

### IVT associated complications in patients with IVT discontinuation

All patients received a cerebral imaging within 24 h after IVT per standard operating procedure (SOP). There were six patients (26.1%) with evidence of ICH after discontinuation, of these two (8.7%) were minor subarachnoidal hemorrhages after EVT. All intracranial hemorrhages are classified in Table 3. None of the ICH led to clinical deterioration and therefore none of the ICH were classified as symptomatic.

Of the patients with relevant extracranial bleedings only one patient needed medical intervention with transfusion of erythrocyte concentrates without need of surgical intervention (BARC type 3a). The other patients with extracranial bleeding complication did not require any specific treatment apart from IVT discontinuation, local compression (if anatomically possible) and monitoring of vital signs according to our stroke SOP (BARC type 2). All extracranial bleeding complications are classified in Table 3. In two patients the IVT was discontinued due to angioedema or allergic reaction, which were treated according to standard operating procedures and the patients recovered from these acute complications.

### Clinical course of patients with IVT discontinuation

The NIHSS score of patients with IVT discontinuation improved during the inpatient stay from median 9 [IQR 4–12] to 3 [IQR 1–7]. Two patients with IVT discontinuation (8.6%) died during the inpatient stay, both due to malignant middle cerebral artery infarction. Both of these patients underwent EVT with successful recanalization of the occluded vessel (mTICI 2b and mTICI 2c).

## Discussion

IVT is the standard therapy for patients with AIS and recently, in addition to alteplase, a second thrombolytic agent, tenecteplase, has become available. The different routes of administration – a 10% bolus followed by a 1-hour infusion of 90% of the dose for alteplase and a single bolus of the total dose for tenecteplase – could offer advantages and disadvantages for both substances in certain situations. The possibility of interrupting or prematurely terminating administration could be an advantage of alteplase in situation where side effects occur or existing contraindications are identified with a delay. Therefore, data on IVT discontinuation in patients with AIS appear to be of great interest for further assessment of clinical relevance.

This real-world data of more than 1,500 IVT patients demonstrate that discontinuation of the rt-PA infusion is rare in clinical practice. Our data showed multiple different reasons, each highlighting a different aspect of the challenges in the time critical treatment of IVT in AIS.

Clinical features like agitation and hypertensive crises led mostly to interruption of IVT with final administration of full dose rt-PA, while allergic reactions, determination of active oral anticoagulation and extracranial bleeding complications led to early termination and incomplete dosing of rt-PA. In two patients with deterioration of neurological symptoms IVT was not restarted after discontinuation during control cerebral imaging to rule out acute intracranial hemorrhage due to long break of IVT. Nevertheless, the administered portion of rt-PA dose was high, at average 61% of the planned dosage in all patients with IVT discontinuation.

IVT was discontinued in five patients due to extracranial bleeding complications, including three patients with spontaneous nasal or oral bleeding and two patients with trauma related bleedings. One patient with trauma related bleeding into arm and hip required a medical intervention (transfusion of erythrocytes due to anemia), while the discontinuation of IVT was late after 85% of the planned total dose of rt-PA in this patient. While data on the bleeding risk of IVT in patients with AIS and minor trauma are scarce, one small study showed an increased risk of bleeding in AIS patients with minor trauma treated with IVT, without affecting the functional outcome [19]. Therefore, the decision for or against IVT in AIS patients with trauma should be made on an individual basis.

The reason for termination of IVT directly after the bolus administration was reinterpretation of the initial acute cerebral imaging in all patients of our study. In all these cases, detection of early signs of larger infarcted areas led to the decision to terminate the IVT. Afterwards, four out of five of the patients with cerebral imaging reinterpretation underwent EVT as a recanalization therapy, which indicates that the use of a different recanalization method may have influenced the decision to stop the IVT and led to the higher ratio of EVT in patients with discontinuation of IVT in our study. However, due to this early termination and therefore low dose of rt-PA administered we cannot provide a definite statement regarding the risk of intracranial hemorrhagic complications with the administration of the full dose IV thrombolytic in these patients. In all of these patients IVT was initiated based on non-contrast CT and it is not possible to determine retrospectively whether an alternative imaging procedure would have influenced the IVT administration or the EVT timing.

No symptomatic intracranial hemorrhage was detected in patients with IVT discontinuation. Besides two patients (8.7%) with asymptomatic hemorrhagic infarction (HI1) and two patients (8.7%) with asymptomatic subarachnoidal hemorrhages after additional treatment with EVT, in two patients (8.7%) asymptomatic parenchymatous hemorrhages (one remote PH, one PH1) were detected. These rates were similar to the rates of radiologically defined intracranial hemorrhages described in the randomized trials of rt-PA IVT for AIS [20]. Both patients with parenchymatous hemorrhages suffered from difficult-to-control arterial hypertension, reflecting the importance of strict monitoring and therapy of blood pressure while and after IVT. These findings are in line with large meta-analysis, which showed an association between high blood pressure and sICH and worse outcome in patients with AIS after IVT [21]. Although all detected ICHs in our study were classified asymptomatic, they should not be interpreted as irrelevant since multiple studies showed worse recovery of patients with asymptomatic ICH [22–25]. 

The frequency of interrupted or stopped rt-PA infusions is not well investigated in literature. In a sub-study of the *Benefits of Stroke Treatment Delivered Using a Mobile Stroke Unit Compared to Standard Management by Emergency Medical Services (BEST-MSU) trial* including 497 patients treated with rt-PA on the Houston Mobile Stroke Unit (MSU) the proportion of interruption or delay of the rt-PA infusion was comparatively high at 8.3% [26]. IVT was mainly stopped for clinical fluctuation and suspected bleeding and problems of administration potentially caused by the specific setting of a mobile stroke unit. IVT was stopped in six patients (1.2%) due to side effects (angioedema) and later detected contraindications (based on cerebral imaging and time-window). Thus, the proportion of IVT discontinuation was identical to our study (1.2%).

There are obvious limitations of our study. Foremost, the number of patients with discontinued IVT was low limiting the statistical power and preventing further subgroup analysis. Due to the small number of patients, complications that occur with a low incidence can only be assessed to a limited extent. Further no analysis for IVT efficacy and functional outcome was available in the full cohort. However, influence of interruption of rt-PA infusion on serum rt-PA concentrations, potentially leading to sub-therapeutic plasma levels, have been demonstrated earlier [27]. In our study the duration of the interruption was not documented. None of the patients received a bolus before restart of the infusion. Further, negative impact of unintentional underdosing of rt-PA for IVT in patients with acute ischemic stroke on functional outcome has been shown in the *Weight Approximation in Stroke Before Thrombolysis (WAIST) study* before [28]. In addition, in the *Enhanced Control of Hypertension and Thrombolysis Stroke Study (ENCHANTED)* the lower dose of 0.6 mg/kg rt-PA compared to standard dose 0.9 mg/kg rt-PA did not meet criteria of non-inferiority [29]. Due to the long investigation time frame multiple minor changes to the SOP in treatment of AIS were implemented in our center, potentially limiting the comparability of the data. However, we did not detect different proportions of discontinued IVT during the course of the study. Altogether, larger multi-centered studies may better describe patients with discontinued thrombolysis and studies directly comparing patients with discontinuation of alteplase IVT and patients in similar clinical conditions treated with tenecteplase IVT, without option of discontinuation, would be desirable.

## Conclusions

Our data demonstrate the rarity of IVT discontinuation in the treatment of AIS, mostly after administration of a relevant dose of rt-PA.

## Supplementary Information

Below is the link to the electronic supplementary material.

Supplementary Material 1

## Data Availability

The data that support the findings of this study are not publicly available due to local regulations but may be available in anonymized form from the corresponding author upon reasonable request and with appropriate permissions.

## Abbreviations

AIS: Acute ischemic stroke
CT: Computed tomography
DISC: Discontinued patients
DTNt: Door-to-needle time
EMA: European medicines agency
ER: Emergency room
EVT: Endovascular treatment
ICH: Intracranial hemorrhage
IQR: Interquartile range
IVT: Intravenous thrombolysis
LVO: Large vessel occlusion
mRS: Modified Rankin Scale
NIHSS: National Institutes of Health Stroke Scale
rt-PA: Alteplase
sICH: Symptomatic intracranial hemorrhage
SOP: Standard operating procedure
STNt: Symptom-to-needle time
TNK: Tenecteplase
