# Supplementary material for: Discontinuation of intravenous thrombolysis in acute ischemic stroke
Source: BMC Neurol. 2026 Oct 9;26:641. doi: 10.1186/s12883-026-05465-5 (PMC13653402; doi:10.1186/s12883-026-05465-5)
Supplement: Supplementary file 1 [file 12883_2026_5465_MOESM1_ESM.docx]

Supplementary Table 1. Blood pressure course and administered medication in patients with discontinuation of IVT due to uncontrolled hypertension after initiation of IVT

| **Type of discontinuation** | **BP at admission** | **BP at IVT start** | **BP at IVT discontinuation** | **Administered i.v.  BP medication** |
| --- | --- | --- | --- | --- |
| Interruption | 187/80 mmHg | 180/100 mmHg | 200/110 mmHg | urapidil, clonidine |
| Interruption | 181/115 mmHg | 105/44 mmHg | 212/116 mmHg | urapidil |
| Termination | 170/110 mmHg | 170/90 mmHg | 200/100 mmHg | urapidil |
